# Changes of Soil Microbiological Properties during Grass Litter Decomposition in Loess Hilly Region, China

**DOI:** 10.3390/ijerph15091797

**Published:** 2018-08-21

**Authors:** Yun Xiang, Shaoshan An, Man Cheng, Lijun Liu, Ying Xie

**Affiliations:** 1 State Key Laboratory of Soil Erosion and Dryland Farming on the Loess Plateau, Northwest A&F University, Yangling 712100, Shaanxi, China; xy020824@163.com (Y.X.); shan@ms.iswc.ac.cn (S.A.); 2 Institute of Loess Plateau, Shanxi University, Taiyuan 030000, Shanxi, China; 3 Shanxi Research Academy of Environment Sciences, Taiyuan 030000, Shanxi, China; lljysd@126.com (L.L.); xieying19926@163.com (Y.X.)

**Keywords:** litter decomposition, soil microbial biomass, soil enzyme activity

## Abstract

Litter, the link between soil and plant, is an important part of nutrient return to soil. Deeply understanding the effect of litter decomposition on soil microbiological properties is important for the sustainable development of grasslands. Three plants (*Thymus quinquecostatus* Celak., *Stipa bungeana* Trin. and *Artemisia sacrorum* ledeb.) leaf litter were selected. A simulation experiment using the nylon bag method was conducted to measure the soil microbial biomass carbon and nitrogen, and soil enzyme activity during litter decomposition. The results showed that the decomposition of three leaf litter enhanced soil microbial carbon and nitrogen. The change rate of soil microbial carbon and nitrogen decreased as Ar.S > St.B > Th.Q. The activities of soil invertase, soil urease, and soil nitrate reductase were significantly improved by the coverage of leaf litter. After 741-day litter decomposition, the change rate of soil invertase was from 16.7% to 33.2%. The change rate of soil urease was highest in the Th.Q treatment; St.B treatment and Ar.S treatment followed, and lowest in the control. The change rates of soil nitrate reductase in the St.B and Ar.S treatment were >1000% higher than those of other treatments. The response of soil enzyme activity to litter decomposition “lagged” behind the change of soil microbial biomass. The significant increase of soil microbial biomass and enzyme activity demonstrated that litter decomposition played an important role in maintaining soil ecological function.

## 1. Introduction

Arid and semi-arid ecosystems account for 30% of the global land area, which plays an important role in ecosystem services [1]. In recent years, with the implementation of a series of ecological projects such as the “Green for Grain Project” and the “Enclosure and Grazing prohibition Project”, soil ecosystems have been restored to varying degrees. An increasing number of studies have confirmed that grassland restoration can significantly promote soil nutrient cycling, organic matter formation, and microbiological properties [2]. Especially at the present stage, with herbivores such as livestock having been withdrawn from the grassland ecosystems, it has been noted that litter is the important “bond” in the soil-plant ecosystems. Litter decomposition is a crucial ecosystem process that determines the accumulation and cycling of C, N, and P between soil and vegetation; it plays an important role in maintaining the material circulation, energy flow, and information transmission [3,4]. Therefore, a deep understanding of litter decomposition and its influences on the soil ecological environment is required to improve our ability to promote the sustainable development of soil and plant compound systems. 

Researchers have conducted more and more studies to explore litter decomposition, which mainly focused on litter decomposition rates [5] and nutrient dynamics [6], soil carbon and nitrogen availability, nutrient cycling and its environmental factors, and so on [7,8,9]. Zhang et al. (2018) showed that the litter decomposition rate is an important major factor affecting soil nutrient dynamics following afforestation [10]. Some studies have further shown that litter quality and quantity influenced both soil microbial biomass and enzyme activity [11,12,13,14], Hu et al. (2005) found that the responses of soil microbiological properties to litter decomposition were much more sensitive than those of soil chemical properties [15]. Although these studies have been conducted in much detail, studies addressing the effects of different plant species on soil microbiological properties are relatively scarce. The research about the characteristics of soil dynamic change during different stages of litter decomposition is still unclear. 

In this study, we hypothesized that the decomposition of grass litter would enhance soil microbial biomass and improve soil enzyme activities. The change characteristics of soil microbial biomass carbon and nitrogen, and the enzyme activities of soil invertase, urease, and nitrate reductase were studied by the nylon−bag method. The study is aimed at evaluating the variation characteristics of soil microbiological properties during litter decomposition, and exploring its correlation with environmental factors and the chemical properties of litter, to deeply understand the function of litter decomposition in semiarid grassland ecosystems and to provide a scientific basis for ensuring the stability and sustainable development of grassland ecosystems.

## 2. Materials and Methods

### 2.1. Study Site

The study was conducted in Shanghuang village of Guyuan City, Ningxia Hui Autonomous Region, China (35°59′−36°03′ N, 106°26′−106°30′ E), which is located in the semiarid hilly area of western Loess Plateau. The altitude of the study region is between 1534 and 1822 m. The study site has a monsoon climate, with a transition from semiarid mid−temperatures to warm temperatures. The region is characterized by a mean annual temperature of 6.9 °C, a mean annual precipitation of 419.1 mm, and an aridity index between 1.55 and 2.00. The total land area of the Shanghuang study area is 7.6 km^2^. The Xiaochuan river divides the study area into two parts: the east and the west. In the east, most of the terrain are beamy hills. In the west, 90% of the terrain in the study area is gully-slope land; 8% is the gentle platform. The types of terrain are, in order, beam, flat, platform from west to east. The studied pedons were classified as Entisols, according to soil taxonomy [16]. The major vegetation types of the study area are artificial shrubs (including *Caragana Korshinskii* Kom., *Prunus davidiana* Franch., and *Armeniaca sibirica* Lam.), artificial grassland (*Medicago sativa* Linn.), abandoned grassland (e.g., *Thymus quinquecostatus* Celak., *Stipa bungeana* Trin., *Artemisia sacrorum* ledeb.), and cropland (mainly including maize, wheat and buckwheat).

### 2.2. Experimental Design

At the end of September 2011, the standing litter of three typical plants including *Thymus quinquecostatus* Celak. (Th.Q)*, Stipa bungeana* Trin. (St.B) and *Artemisia sacrorum* ledeb. (Ar.S), were randomly collected from abandoned grassland in the Shanghuang study area. The impurities adhering to the litter surface were brushed out with a soft brush. The litter samples were fixed at 105 °C for 30 min, and then oven-dried at 65 °C to obtain a constant weight. Part of the samples were crushed to sieve through a 1 mm × 1 mm sieve, and the initial chemical properties were measured subsequently (the results are shown in Table 1). The total carbon (TC) of litter was measured by the potassium dichromate oxidation method, and total nitrogen (TN) by the Kjeldahl method [17]. The lignin of litter was determined using the acid−detergent digestion technique washing method, as suggested by Van Soest (1963) [18], and cellulose as suggested by Van Soest (1967) [19]. The rest of the samples were stored in kraft paper bags. 

In this study, the plant litter decomposition was simulated by the nylon-bag method. In May 2014, a typical piece of abandoned land was selected as the study plot, and the plants and their residues were cleaned up. Then the study plot was divided by PVC plates into several subplots of 30 cm × 30 cm as the decomposition area. An iron fence was set up around the plot to prevent interference from human and animals. The soils of these subplots were basically the same. There were three litter treatments (i.e., Th.Q, St.B, and Ar.S treatments) and one control treatment; all treatments were replicated three times.

In each litter treatment, 40 g litter was placed into a nylon bag with a mesh size of 0.05 mm × 0.05 mm, which was attached to the soil surface. In the control treatment, an empty nylon bag was placed on the soil surface. The experiment began on 12 May 2014, and finished on 17 May 2016, lasting a total of 741 days. Due to the subzero temperature in the experimental area in winter (from November to March), samples were not collected in this period. A destructive sampling method was used 9 times, for a total of 108 subplots. During the incubation period, soil was sampled from the 0–5 cm soil layer below the nylon bags on 108th, 177th, 323rd, 375th, 437th, 479th, 532nd, 692nd, and 741st days. Then the animal and plant residues were removed. Some samples were stored at −20 °C, others were air dried, ground, sieved, and stored for the soil nutrient determination.

**Figure 1:**
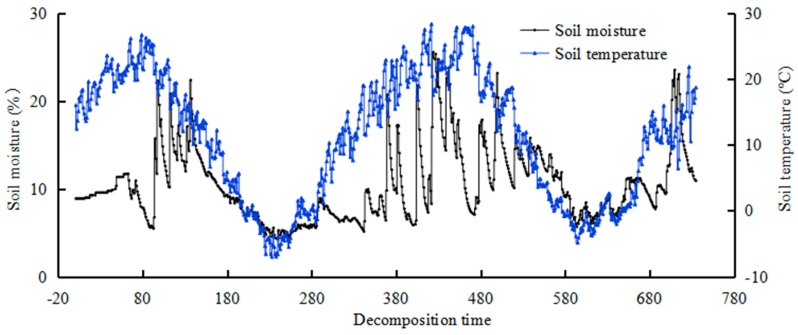
Soil moisture content and the temperature during litter decomposition process.

In order to better illuminate the influencing factors of soil nutrient change in the decomposition process, nine TDT probes (Time Domain Trans-missometry; Acclima, Inc., Meridian, ID, USA) were installed in the subplots to monitor changes of soil temperature and moisture in the decomposition area. The probes were horizontally buried at 5 cm above the surface, which represents the temperature and soil volumetric water content at 5 cm in the decomposition area, to simultaneously monitor the temperature and moisture once every hour, i.e., 24 times per day, during the whole decomposition process. The soil moisture content and temperature during the field experiment are shown in Figure 1.

### 2.3. The Determination of Soil Microbial Biomass and Enzyme Activity

Soil microbial biomass carbon (MBC) and nitrogen (MBN) was measured using the chloroform fumigation-extraction method. MBC was determined by a TOC analyser (Phoenix 8000, Tekmar Dohrman, Mason, OH, USA), and MBN was determined colorimetrically with a spectrophotometer (Hitachi, Tokyo, Japan, UV2300) at 220 nm and 275 nm. The MBC and MBN were calculated using a *K*_C_ factor of 0.38 and a *K*_N_ factor of 0.45, respectively [20].

The activity of soil invertase, urease, and nitrate reductase was determined as described [21]. Soil invertase activity was measured by the method of 3,5-dinitrosalicylic acid colorimetry using sucrose as the substrate. The released glucose was quantified colorimetrically with a spectrophotometer (Hitachi, Tokyo, Japan, UV2300) at 508 nm, and results expressed as mg glucose g^−1^ 24 h^−1^. Soil urease activity was measured using urea as the substrate after 24-h incubation at 37 °C. The activity of soil urease was determined using a spectrophotometer (Hitachi, Tokyo, Japan, UV2300) at a wavelength of 578 nm, with results expressed as mg NH_4_ g^−1^ 24 h^−1^. Soil nitrate reductase activity was determined using potassium nitrate as the substrate. The released nitrite nitrogen was assayed colorimetrically at 520 nm, and the results expressed as μg NO_2_ g^−1^ 24 h^−1^.

### 2.4. Data Analysis

To better investigate the effects of litter decomposition on soil microbial biomass and enzyme activity, the change rates of soil microbial biomass and enzyme activity were calculated according to the following formula:

Δ*P* (%) = (*P*_741d_ − *P*_0d_)/*P*_0d_

where *P* represents the indicators involved in soil microbial biomass carbon and others; *P*_741d_ represents the soil microbial carbon and other indicators at the 741st day of decomposition; *P*_0d_ represents the initial soil microbial biomass carbon and other indicators.

All data in the experiment were calculated via Microsoft Excel version 2003 and SPSS version 18.0. The value of each indicator was expressed by mean ± standard deviation. The change rates of soil microbiological properties from different plant litter treatments were compared using the S-N-K method. The relationship between the change rates of soil microbial properties and the initial chemical properties of litter was evaluated by Pearson correlation analysis. The comprehensive influence of litter decomposition on soil microbiological properties were evaluated using principal component analysis (PCA) via Canoco 5.0. (Microcomputer Power, Ithaca, NY, USA). The data involved soil MBC, MBN, invertase, urease, nitrate reductase, soil moisture and soil temperature during litter decomposition. 

## 3. Results

### 3.1. Change Characteristics of Soil Microbial Biomass

**Figure 2:**
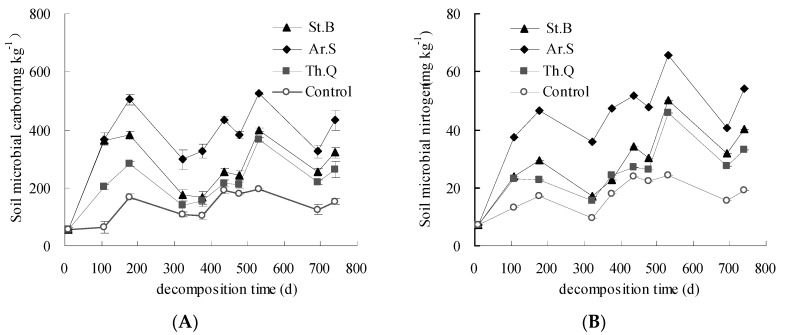
The change characteristics of soil microbial biomass carbon (**A**) and nitrogen (**B**).

The change characteristics of soil microbial biomass carbon and nitrogen in field litter decomposition are shown in Figure 2. The contents of soil microbial biomass carbon among plant litter treatments and control treatment showed basically the same trend, but the contents of litter treatments were all higher than those of control. For the three litter treatments, the contents of soil microbial biomass carbon in 0–5 cm soil layer were between 56.9 and 526.5 mg kg^−1^ (Figure 2A). It can be found that the microbial biomass carbon content of Ar.S was the highest, St.B was second, and Th.Q was relatively lower. On the 741st day, the soil microbial biomass carbon contents of Ar.S, St.B, and Th.Q in 0–5 cm soil layer were 180.2, 168.82, and 110.8 mg kg^−1^ higher than that of the control, respectively. The contents of soil microbial biomass nitrogen of the control in 0–5 cm soil layer were between 7.1 and 24.4 mg kg^−1^ (Figure 2B). The contents of soil microbial biomass nitrogen of litter treatments were all higher than that of the control, i.e., in 0–5 cm soil layer were between 7.1 mg kg^−1^ and 65.8 mg kg^−1^. On the 741st day, the soil microbial biomass nitrogen levels of three litter treatments were all higher than that of the control. The soil microbial biomass nitrogen level of Ar.S was 30.0 mg kg^−1^ higher than that of the control. 

### 3.2. Change Characteristics of Soil Enzyme Activities

**Figure 3:**
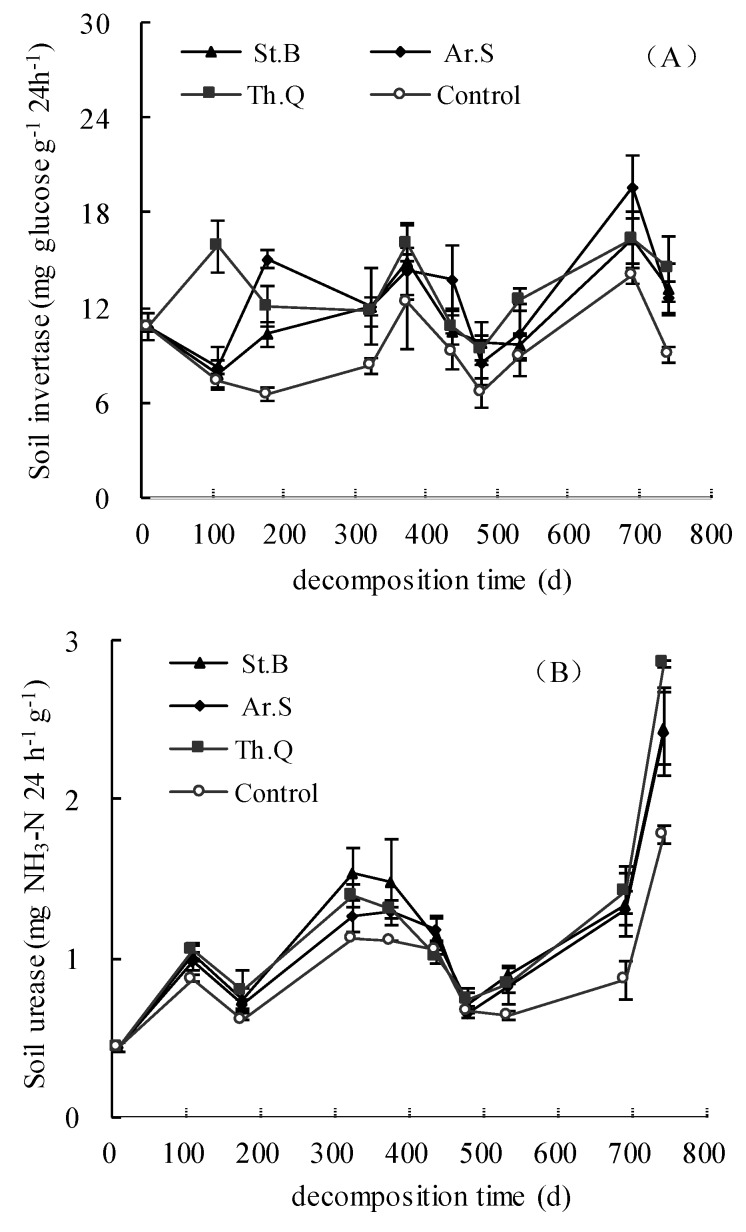

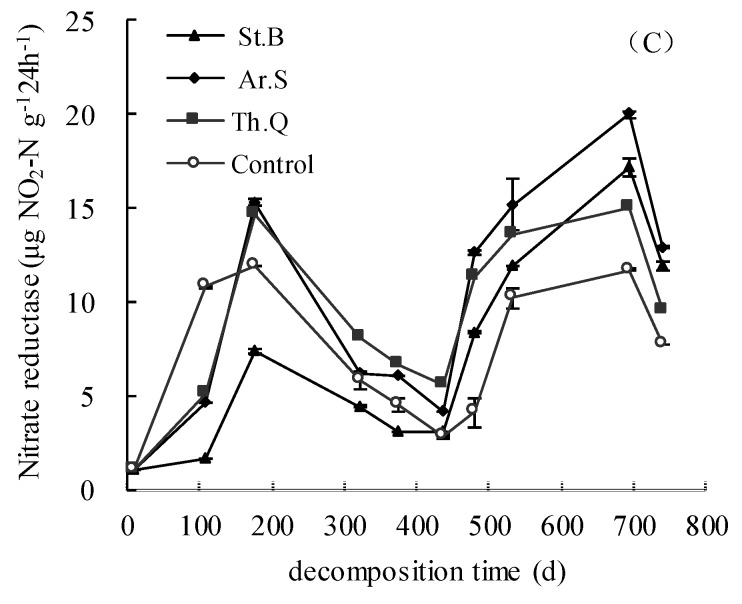
The change characteristics of the activities of soil invertase (**A**), soil urease (**B**) and soil nitrate reductase (**C**).

As shown in Figure 3A, in the control treatment, soil invertase activity in 0–5 cm soil layer was between 6.66 and 14.07 mg glucose g^−1^ 24 h^−1^; the minimum value appeared on the 479th day, the maximum value appeared on the 692nd day. The values of soil invertase activity of litter treatments in 0–5 cm soil layer were between 7.86 and 19.6 mg glucose g^−1^ 24 h^−1^. On the 741st day, the soil invertase activity of Th.M, St.B, and Ar.S were 4.13, 3.60, 5.39 mg glucose g^−1^ 24 h^−1^ higher than that of the control, respectively. Before the 323rd day, the soil invertase activity among different plant litter treatments and the control showed a different trend, but after that, the trend was basically the same.

As shown in Figure 3B, in the control, the values of soil urease activity in the 0–5 cm soil layer were between 0.43 and 1.78 mg NH_3_−N g^−1^ 24 h^−1^. The values of soil urease activity from the leaf litter treatments in the 0–5 cm soil layer were between 0.43 and 2.86 mg NH_3_−N g^−1^ 24 h^−1^. The soil urease activity values among plant litter treatments and CK showed a similar trend: rise-decline-rise-decline-rise. On the 741th day, in the 0−5 cm soil layer, comparing with the control, the values of soil urease activity of Th.M, St.B, and Ar.S were significantly increased by 37.4%, 36.2%, and 60.4%, respectively.

The soil nitrate reductase activity in 0−5 cm soil layer was between 1.02 and 11.89 μg NO_2_−N g^−1^ 24 h^−1^. During the leaf litter decomposition, the variation trend of soil nitrate reductase activity was rise (before the 177th days)-decline (from the 177th to 437th day)-rise (from the 437th to 692nd days)-decline (from the 692nd to 741st day). The soil nitrate reductase activity among the litter treatments and control had basically the same trend, but the effects of different plant litter treatments on soil nitrate reductase activity were different. In the 0–5 cm soil layer, before the 108th day, the soil nitrate reductase of Ar.S and Th.M were lower than those of control; from the 175th to the 437th day, the values of Ar.S and Th.M were both higher than those of control; after the 437th day, the soil nitrate reductase of three litter treatments were higher than that of control.

### 3.3. The Change Rate of Soil Microbial Biomass and Enzyme Activity

Table 2 shows the change rates of soil microbial biomass and enzyme activity after a 741-day decomposition. Except soil invertase activity, the change rates of other soil biological properties were all higher than 100%, which indicated that soil microbial biomass and enzyme activity were very sensitive to litter decomposition. The change rates of soil microbial biomass and enzyme activity were significantly higher than those of the control. The change rates of soil microbial biomass carbon and nitrogen decreased in this order: Ar.S > St.B > Th.M > control. The change rates of soil invertase activity of litter treatments were from 16.7% to 33.2%. The change rate of soil urease was highest in the Th.Q treatment, followed by the St.B treatment, and then Ar.S treatment; it was lowest in the control treatment. The change rate of soil nitrate reductase in the control was as high as 650%. The change rates of soil nitrate reductase among litter treatments were significantly higher than that of control; among them, the soil nitrate reductase of Ar.S and St.B treatments were both significantly higher than that of Th.Q treatment.

### 3.4. The Pearson Correlation between the Environmental Factor, Substance and Soil Biological Properties

Table 3 shows the Pearson correlation coefficients among the indicators of soil biological properties, environmental factors, and litter initial chemical properties. Soil invertase activity had a significant negative correlation to the total carbon (*p* < 0.01), whereas had a significant positive relation with lignin/N of leaf litter during the litter decomposition (*p* < 0.01). The soil nitrate reductase activity had a very significant positive correlation to total carbon of leaf litter (*p* < 0.01), and a significant negative correlation to lignin/nitrogen of leaf litter (*p* < 0.01).

**Figure 4:**
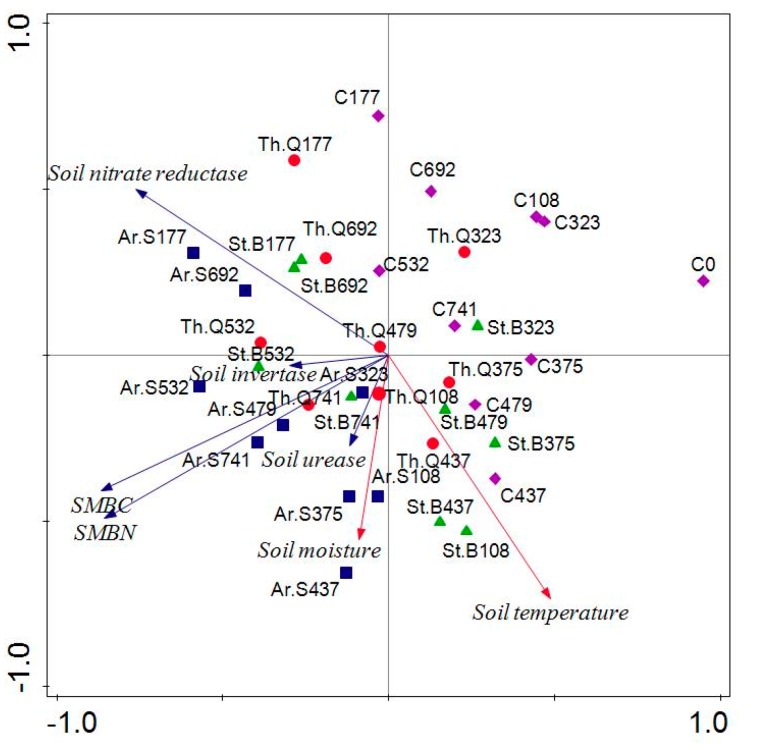
Principal component analysis of soil moisture and temperature and microbial properties. Note: Soils were named after litter type plus decomposition time.

The results of PCA of soil moisture and temperature and microbial properties are shown in Figure 4. The contribution of PC1 and PC2 accounted for 80.2%. There was a positive correlation among soil invertase, SMBC, SMBN, Soil urease, and soil moisture. The soils from different treatments were observed in different quadrants. The soils from the control were observed in the first quadrant. The soils from the Ar.S treatment were found in the second and third quadrant, while, as expected, the Th.Q323 and St.B323, the soils from the Th.Q and St.B treatment were distributed in the second, third, and fourth quadrants. This indicated that soil microbiological properties have changed significantly.

## 4. Discussion

### 4.1. Effects of Leaf Litter Decomposition on Soil Microbial Biomass

Soil microbial biomass is the most active component of soil organic matter, directly or indirectly regulate and control the transformation and supply of soil nutrients, and closely related to the transformation of soil organic matter, nitrogen and other nutrients [22]. Our results showed that the decomposition of grass leaf litter can significantly enhance the content of soil microbial biomass carbon and nitrogen. This is consistent with part of previous studies. Liu et al. (2012) found that in the coniferous forestland of loess area, the application of Chinese pine litters resulted in increasing soil microbial biomasses [23]. Many studies have shown that the removal of surface litter can result in a significant reduction in soil microbial biomass [24]. However, some studies have shown that the removal or addition of litters had no significant effect on the soil microbial biomass. Fisk and Fahey (2001) found that soil microbial carbon did not show significant difference between control and fresh litter removal treatments after an 8-year removal of leaf litter in a temperate deciduous forest [25]. Prévost−Bouré et al. (2011) conducted a one-year study on the removal of litter, control, and double litter treatments in a mature oak forest in France, and found no significant difference in soil microbial biomass among the three treatments [26]. Matsushima and Chang (2007) studied on a 13a Spruce forest in Canada, found that the removal of litter did not cause changes in the soil microbial biomass nitrogen content of surface soil [27]. The different consequences of the field simulation experiments are due to the test methods and the selection of the test plots. In previous studies, the litter removal and addition test were mainly carried out in the tropical forest, the elements such as root growth were still imported to the woodland, the long-term removal of litter would make the soil microorganisms adapt to the state of non-litter input and spontaneously adjust its community structure, and also the root growth had a positive feedback to soil microbial biomass. This experiment was carried out in Loess hilly region, choosing the abandoned land as the plot, establishing the subplot with the removal of all plants on the ground, and assuming that the soil was the same of all the subplots. In the decomposition process, except for the leaf litter, there was very little input of organic matter from the outside. During the leaf litter decomposition, the soluble matter in litter leaves was leached into the surface soil to promote the propagation and growth of microorganisms.

### 4.2. Effects of Litter Decomposition on Soil Enzyme Activities

Soil enzyme activities are associated with the cycle of the relevant elements in the soil, which can reflect the intensity and direction of soil biochemical reactions, and plays an important role in the process of soil material cycling and energy conversion [28]. The results of litterbag experiment showed that the activities of soil invertase, soil urease, and soil nitrate reductase were all improved by the coverage of plant litter significantly. Among them, the increase of soil urease activities and soil nitrate reductase activities both mainly occurred in the late stage decomposition, namely after the 532nd day. Hu et al. (2005) used field simulation experiment to find that the decomposition of alder litter could significantly increase the activities of soil urease and soil invertase [15]. Gray (2002) suggested that the soil enzyme activity was significantly increased when the plant residue was applied to the soil [13]. Lu et al. (2008) compared the remove litter plots and kept litter plots to research the response of the soil properties of the Caribbean pine forest to the removal of litter; after 5 years, the results showed that soil urease, phosphatase and catalase decreased by 35%, 49%, and 49%, respectively, compared with the control [29]. Liu et al. (2012) selected the coniferous pure forest of loess gulled-hilly area as the study site, found that the addition of Chinese pine litter lead to the decrease of soil urease and invertase; the addition of platycladus orientalis lead to the increase of soil invertase, and the decrease of soil urease [23]. Soil enzyme is a bioactive protein in soil, which has a strong specificity. The differences among these studies are probably because soil enzyme activities are very susceptible and comparatively complex [30]. Soil enzymes mainly come from the process of microbial metabolism [15]. In our study, the different effects of three litter types on soil enzyme activities are mainly because of the differences among the types of exogenous litter, and the differences among soil microbial biomass, microbial communities, and metabolic processes which resulted from different litters [13]. According to the characteristics of soil microorganism and enzyme activities, we also found that the change of soil enzyme in time “lagged” behind soil microbial biomass. This is in agreement with the results of Hu et al. (2005) [15]. The dis-syenrgized increasing of soil microbial biomass and enzyme activity might result from the changes of soil microbial community structure [31]. Therefore, further studies are needed to better understand the mechanistic link between soil enzyme and soil microbial communities during litter decomposition.

### 4.3. Factors Influencing Soil Microbiological Properties

Litter decomposition can be completed under the synthetical action of litter and soil microorganisms. The long-term decomposition of litter was dominated by carbon/nitrogen ratio, lignin/nitrogen ratio, etc. [32]. We found that litter carbon and lignin/N was the most important factor affecting soil intertase and nitrate reductase activities, indicating that the litter chemistry regulated the soil enzyme activity. Hu (2005) indicated that the chemical properties of litter may be the intrinsic factor which determines the effect of litter on soil [15]. Güsewell and Freeman (2005) and Warning et al. (2013) suggested that species’ identity, associated with litter nutrient availability, had a great influence on enzyme activity, and further on litter decomposition rate [33]. Ge et al. 2017 found that soil enzymes differed in their responses to litter N content during litter decomposition [34]. Moreover, we found that soil moisture was the major abiotic factor which had a positive relation with SMBC (soil microbial biomass carbon), SMBN (soil microbial biomass nitrogen), soil invertase, and soil urease. Schimel et al. suggested that long term drying resulted in the decreasing of soil microbial biomass and respiration and the change of soil microbial community structure [35]. In short, we can conclude that, in semiarid grassland, soil microbiological properties was determined by the litter carbon and lignin/N associated with soil moisture.

## 5. Conclusions

In this paper, by the field simulation experiment of litter decomposition of *Artemisia sacrorum*, *Stipa bungeana* and *Thymus mongolicus*, we found that the decomposition of three plant litter could improve soil microbiological. properties. Different leaf litters had different effects on soil microbial biomass and enzyme activity. Soil moisture, litter C, and lignin/N were the most important factors determining the changes of soil microbiological properties. The increase of soil microbial biomass and enzyme activity demonstrated that the litter decomposition plays an important role in maintaining soil ecological function. Therefore, in the follow-up management of semiarid grassland vegetation restoration, the interference of human factors should be minimized to ensure the stability of the litter layer and maintain soil ecological environment.

## Figures and Tables

**Table 1:** The chemical properties of tested leaf litters.

Leaf Litter Types	Total C/g·kg^−1^	Total N/g·kg^−1^	Lignin/%	Cellulose/%	Lignin/N	C/N
St.B	479.55 ± 1.64a	10.58 ± 1.09b	26.84 ± 0.79b	16.00 ± 0.07b	2.5	43.1
Ar.S	481.05 ± 9.39a	17.69 ± 1.15a	30.34 ± 0.34a	8.15 ± 0.12c	1.7	28.2
Th.Q	476.34 ± 22.71a	10.42 ± 1.12b	39.39 ± 0.99a	21.83 ± 0.14a	3.8	48.2

Note: Different lowercase letters mean a significant difference in the chemical properties of different plant litters.

**Table 2:** The indicators of soil microbial biomass and enzyme activities during litter decomposition.

Litter Type	ΔSMBC (%)	△SMBN (%)	ΔSoil Invertase (%)	ΔSoil Urease (%)	ΔNitrate Reductase (%)
St.B	463.7 ± 10.3b	453.7 ± 12.5b	21.6 ± 15.2b	463.6 ± 21.3b	1066.1 ± 35.2a
Ar.S	659.2 ± 20.5a	650.2 ± 20.1a	16.7 ± 8.7b	459.1 ± 19.6b	1166.3 ± 39.6a
Th.Q	361.8 ± 15.6c	351.8 ± 10.2c	33.2 ± 8.9a	558.3 ± 20.1a	832.7 ± 34.5b
Control	167.3 ± 10.0d	169.5 ± 10.1d	−16.4 ± 8.9c	310.3 ± 19.8c	658.3 ± 21.6c

Note: Different lowercase letters mean significant difference in the indicators of soil biological properties in different plant litter or root decomposition.

**Table 3:** Pearson correlation coefficients between the initial chemical properties of litter and the indicators of soil biological properties.

Pearson Correlation Coefficients	The Initial Chemical Properties of Litter					
	TC	TN	Lignin	Cellulose	Lignin/N	C/N
ΔMBC (%)	0.924	0.949	−0.561	−0.995	−0.950	−0.964
ΔMBN (%)	0.923	0.965	−0.560	−0.995	−0.961	−0.948
ΔSoil invertase (%)	−0.999 **	−0.745	0.844	0.951	0.996 **	0.742
ΔSoil urease (%)	−0.964	−0.555	0.952	0.843	0.940	0.550
ΔNitrate reductase (%)	0.999 **	0.748	−0.842	−0.952	−0.997 **	−0.744

Note: ** correlation is significant at the 0.01 level, TC: total carbon, TN: total nitrogen, MBC: microbial biomass carbon, MBN: microbial biomass nitrogen.
